# The Complexity of Weak Rhesus Positivity in Pregnancy: Challenges and Management

**DOI:** 10.5811/cpcem.53965

**Published:** 2026-04-07

**Authors:** Vimoltip Luksanapisitakul, Anas Alojayli

**Affiliations:** London North West University Healthcare NHS Trust, Department of Obstetric and Gynaecology, London, Borough of Brent, United Kingdom

Dear Editor:

We read with great interest “The Complexity of Weak Rhesus Positivity in Pregnancy: Challenges and Management--A Case Report,” which was published in *Clinical Practice and Cases in Emergency Medicine*, Volume 9 Issue 4.

We had a recent case in our maternity unit that shared similarities with the one reported above in its presentation and highlights the recurring challenges faced in clinical management in emergency contexts. A 37-year-old gravida four para three woman presented with second trimester vaginal bleeding to our hospital. During her three previous pregnancies in Canada, she had been classified as Rhesus (Rh)-positive and had not received any Rh immune globulin (RhIG) prophylaxis. In this pregnancy, in her first trimester, group and screen blood demonstrated a weak D reaction, and her Rh type was initially reported as Rh-positive. The sample was subsequently sent for further analysis in accordance with protocol, which confirmed a partial-D, and her status was later amended to Rh-negative. Identification of variant D blood type is clinically significant in obstetrical care, as these patients remain at risk of developing anti-D antibody, which can lead to the potentially severe and sometimes fatal complication of hemolytic disease of the fetus and newborn.

According to the Royal College of Obstetrics and Gynaecology *Green-top Guideline No. 63: Antepartum Haemorrhage*, RhIG prophylaxis should be administered to all non-sensitised Rh-negative women following any potential sensitising event, including second-trimester vaginal bleeding, and this should be given within 72 hours to minimise the risk of alloimmunization.^1^ This aligns with the recommendations of the British Committee for Standards in Haematology, which advise that a maternal blood group and antibody screen should be tested to establish or confirm Rh status and to detect the presence of immune anti-D.^2^ The Committee also recommends that women with anomalous or indeterminate Rh typing status should be managed as Rh-D negative until definitive confirmatory testing has been completed.^2^

Understandably, this patient was perplexed and concerned regarding the advice to receive RhIG, particularly as she had not been offered this in her previous pregnancies. Approximately 15% of the British population are Rh-D negative, which means their red blood cells lack a protein called the Rh-D antigen on their surface.^3^ Although precise data on the prevalence of Rh-D variant are under-documented, estimates suggest a frequency of 0.2–1% among White populations.^4^ Despite the relatively low prevalence, individuals with Rh-D variant may present substantial clinical challenges, particularly when discrepant laboratory findings occur or when urgent management is required in emergency settings.

Although differing in context, both cases highlight the challenges associated with managing variant D blood groups including weak D and partial D in emergency settings. The implementation of globally standardised laboratory testing for variant D antigens is essential, as it plays a critical role in guiding the safe and effective administration of RhIG and ensuring appropriate clinical management, particularly in urgent scenarios.
